# Synergistic effect of chlorogenic acid and levofloxacin against *Klebsiella pneumonia* infection in vitro and in vivo

**DOI:** 10.1038/s41598-020-76895-5

**Published:** 2020-11-17

**Authors:** Shirui Tan, Jing Gao, Qingrong Li, Tieying Guo, Xiangshu Dong, Xuehui Bai, Jinghui Yang, Shumei Hao, Feifei He

**Affiliations:** 1 School of Agriculture, Chenggong Campus, Yunnan University, South Section, East Outer Ring Road, Chenggong District, Kunming, 650500 People’s Republic of China; 2 Center for Life Sciences, School of Life Sciences, Yunnan University, Kunming, 650500 People’s Republic of China; 3 Dehong Tropical Agriculture Research Institute of Yunnan, Ruili, 678600 People’s Republic of China; 4 School of Life Sciences, Yunnan Normal University, No.1, Yuhua Area, Chenggong District, Kunming, 650500 Yunnan People’s Republic of China; 5 The Second Affiliated Hospital of Kunming Medical University, Kunming, 650101 People’s Republic of China; 6 Department of Paediatrics, The First People’s Hospital of Yunnan Province, The Affiliated Hospital of Kunming University of Science and Technology, 157 Jinbi Road, Kunming, 650032 People’s Republic of China; 7 Yunnan Clinical Medical Center for Hematological Diseases, The First People’s Hospital of Yunnan Province, 157 Jinbi Road, Kunming, 650032 People’s Republic of China

**Keywords:** Biochemistry, Chemical biology, Drug discovery, Molecular biology, Diseases

## Abstract

The study aimed to investigate the antibacterial effect and potential mechanisms of chlorogenic acid (CA) in *Klebsiella pneumonia* (KPN) induced infection in vitro and in vivo. 62 KPN strains were collected from the First People’s Hospital of Yunnan Province. CA and CA combined Levofloxacin (LFX) were detected for KPN biofilm (BF) formation in vitro. The lung infection mice model were established by KPN. The effect of CA (500 mg/kg), LFX (50 mg/kg) and CA combined LFX (250 mg/kg + 25 mg/kg) were evaluated through the survival of mice, the changes of inflammation factors of tumor necrosis factor-alpha (TNF-*α*), interleukin (IL)-1*β* and IL-6 in serum, the histopathological analysis of lung and the protein expression of NLRP3 signaling pathway in vivo. A total of 62 KPNs were isolated and identified, of which 13 (21%) strains were BF positive. 8 (13%) strains were extended spectrum *β*-lactamase strains (ESBLs), and 20 (32%) strains are ESBLs biofilm positive. In vitro study, CA and LFX showed a synergistic effect on KPN biofilm formation. In vivo mice experiment, CA, especially CA + LFX treated group significantly decreased the serum levels of TNF-*α*, IL-1*β* and IL-6, improved the survival ratio and lung pathology changes, and also reduced the protein expression of ASC, caspase 1 p20, IL-1*β* and phosphor NF-κB p65. CA could effectively alleviate lung infection of KPN infected mice, and the antibacterial effection is strengthened by combined with LFX. The study provide a theroy basis for making rational and scientific antibacterial therapy strategy in clinic.

## Introduction

In recent years, infectious diseases caused by antibiotic resistant bacteria have brought great difficulties to clinical treatment, and have a significant impact on public health and society^1,2^. *Klebsiella pneumonia* (KPN) is the most common clinical pathogens, and is also one of the important pathogens of hospital infection^3^. KPN is divided into three subspecies: pneumoniae subspecies, rhinoscleromatis subspecies and ozaenae subspecies. KPN belongs to human intestinal microflora, which is a conditional pathogen. It is widely distributed in nature, mainly in human respiratory tract, urinary tract, gastrointestinal tract and other parts^4–6^. Under normal conditions, KPN does not cause disease. While when the organism's immunity decreases lead to the imbalance of flora, it will cause respiratory, digestion and urinary tract infections^7–9^. KPN is the second only to *Escherichia* coli in the blood infection pathogens, and has a high mortality rate for carbapenems resistant KPN induced blood infection. KPN is a common bacteria in clinic, which not only cause primary pneumonia, but also meningitis, peritonitis, traumatic infection, septicemia and urinary infection^10–12^.

KPN is easy to produce biofilm (BF), which can encapsulate bacteria layer by layer and increase the bacterial tolerance to antibiotics^13^. With the development of BF research, it is found that KPN resistance to antibiotics is closely related to BF formation, and the main component of BF is polysaccharide protein complex^14^. The research at home and abroad shows that the combination of quinolones and macrolides can effectively inhibit the synthesis of polysaccharide protein complex by bacteria, thus inhibiting the formation of BF^15^. In recent years, the emergence of carbapenem resistant enterobacteriaceae (CRE) bacteria has brought new problems to the treatment of clinical infectious diseases, especially the carbapenem resistant *Klebsiella pneumoniae* (CRKPN), which is increasing year by year^16^. The isolation rate of KPN increased year by year, and the drug resistance rate increased year by year. With the irrational use of antibiotics, KPN resistance is becoming more and more serious. Multi drug resistance, extensive drug resistance and pan drug resistance are not rare^17^. In 2017, the detection rate of CRKPN in China increased to 9%^16^. CRKPN remain sensitive to myxomycetin and tegacyclin in vitro, and it is resistant to most of the other antimicrobial drugs, which leads to more limited for the therapy options^18,19^. Therefore, CRKPN causes serious infection and high mortality, which attracts more and more attention all over the world.

**Figure 1:**
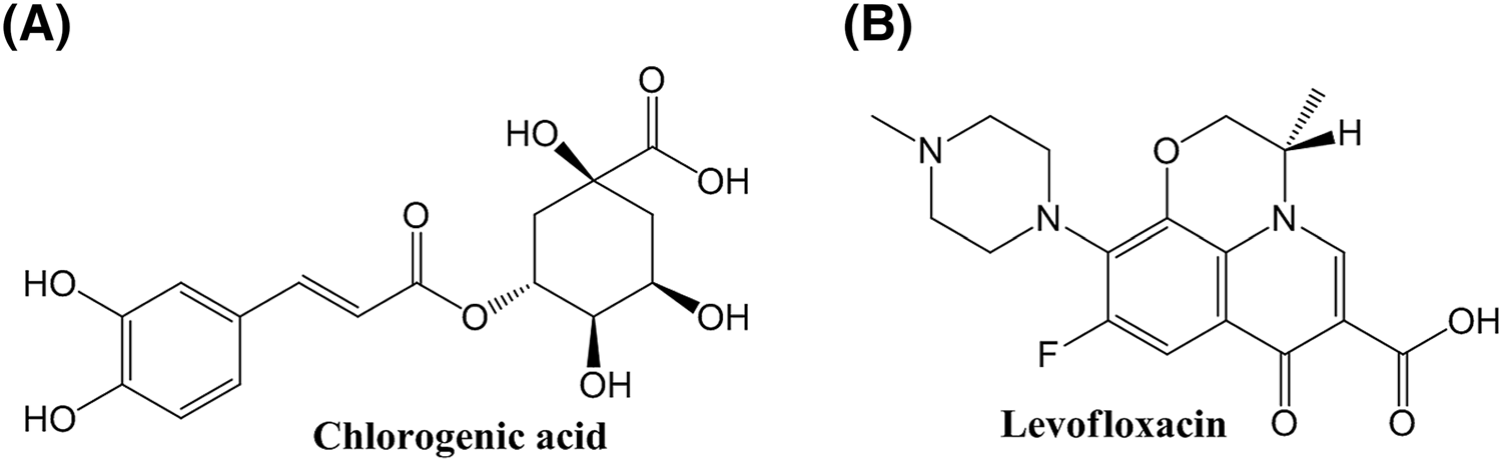
Chemical structures of small molecules. (**A**) Chlorogenic acid, (**B**) Levofloxacin.

Chlorogenic acid (CA), mainly extracted from natural plants, is a kind of phenylpropanoid compound produced by shikimic acid pathway during aerobic respiration of plants (Fig. 1A)^20^. It is synthesized by the condensation of caffeic acid and quinic acid^21^. CA is an important active component in many edible plants and medicinal plants, which is widely distributed in plants, from high dicotyledons to ferns, but there are few plants with high content^22^. CA is mainly in the plant family of Eucommia, Lonicerae and Artemisia, including eucommia, honeysuckle, artemisia, artemisia sunflowers, relay flowers, coffee and cocoa trees^23^. According to the research of Gao et al.^24^ and Huang et al.^25^, CA can alleviate dextran sodium sulfate-induced ulcerative colitis, and benign prostatic hyperplasia in mice through the functions of scavenging free radicals^26,27^, anti-lipid peroxidation^28^, anti-bacterial and antiviral^29^. It has been shown that CA can inhibit the biofilm formation of *Pseudomonas aeruginosa*, but the inhibition effect of CA on KPN is not reported.

Levofloxacin (LFX) is the third generation of fluoroquinolones developed by Japan first pharmaceutical Co., Ltd. in 1986 (Fig. 1B)^30^. It is the L-optically active isomer of ofloxacin, which has the antibacterial activity about twice of that of ofloxacin^31^. The main mechanism of LFX is to inhibit the DNA rotation enzyme and hinder the DNA replication of bacteria^32^. LFX has a wide antibacterial spectrum, high bioavailability and good oral absorption, however, with the frequent use and unreasonable use of LFX, the resistance rate of KPN to LFX increases year by year^33–35^.

Thus, in this study, we mainly aimed to explore and investigate the antibacterial effect and potential mechanisms of CA and the combination of CA and LFX on *Klebsiella pneumonia* (KPN) in vitro and in vivo animals, which can be provide the experimental evidence for further clinical therapy of KPN infection diseases.

## Results

### ESBLs and BF strains identification from 62 strains of KPN

**Figure 2:**
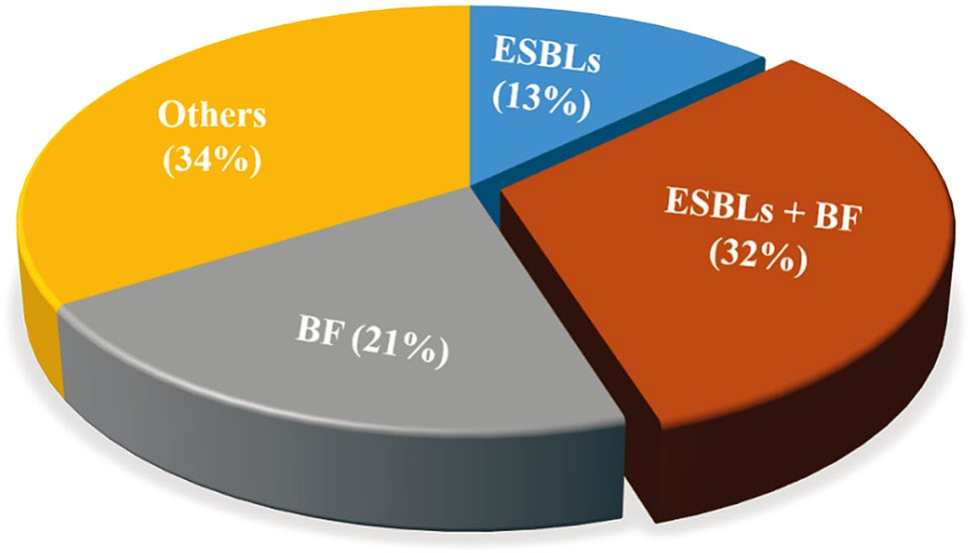
Composition of ESBLs strains, BF strains and ESBLs + BF strains in 62 strains of KPN.

After the identification of the ESBLs and BF strains 62 strains of KPN, we found that among the 62 strains of KPN, 8 strains were ESBLs (13%), 13 strains were BF (21%), and 20 strains were ESBLs and BF (32%), as shown in Fig. 2.

In addition, we further found three strong ESBLs biofilm positive strains (marked as strain A, B and C) from the 20 strains of ESBLs biofilm positive strains through crystal violet staining. Then we detected the MIC values of CA, LFX, and CA + LFX in these three strains. The result showed that combination of CA and LFX obviously increased the bacteria inhibition effect, compared with single administration of CA, which showed a synergistic effect for CA and LFX. Details showed in Table 1.

**Table 1:** Antibacterial effect of CA, LFX and CA + LFX.

Group	MIC for single drug (MICs, μg/mL)		MIC for drug combination (MICc, μg/mL)		
	CA	LFX	CA	LFX	FICI
Strain A	2047.96 ± 3.70	31.93 ± 3.24	512	8	0.25
Strain B	2047.78 ± 5.43	64.02 ± 7.15	512	16	0.16
Strain C	2048.22 ± 6.31	32.11 ± 2.84	512	8	0.25

Data are expressed as mean ± SD for each group, the experiment was repeated by three independent experiments. *MIC* Minimal inhibitory concentration, *CA* Chlorogenic acid, *LFX* Levofloxacin, *FICI* Fractional inhibitory concentration index. FICI ≤ 0.5 indicates synergy, FICI > 0.5–4.0 indicates no interaction, FICI > 4.0 indicates antagonism. Strain A, B and C are the three strongest ESBLs biofilm positive strains from the 20 strains of ESBLs biofilm positive strains.

### Effect of drug combination of CA and LFX on biofilm formation

**Figure 3:**
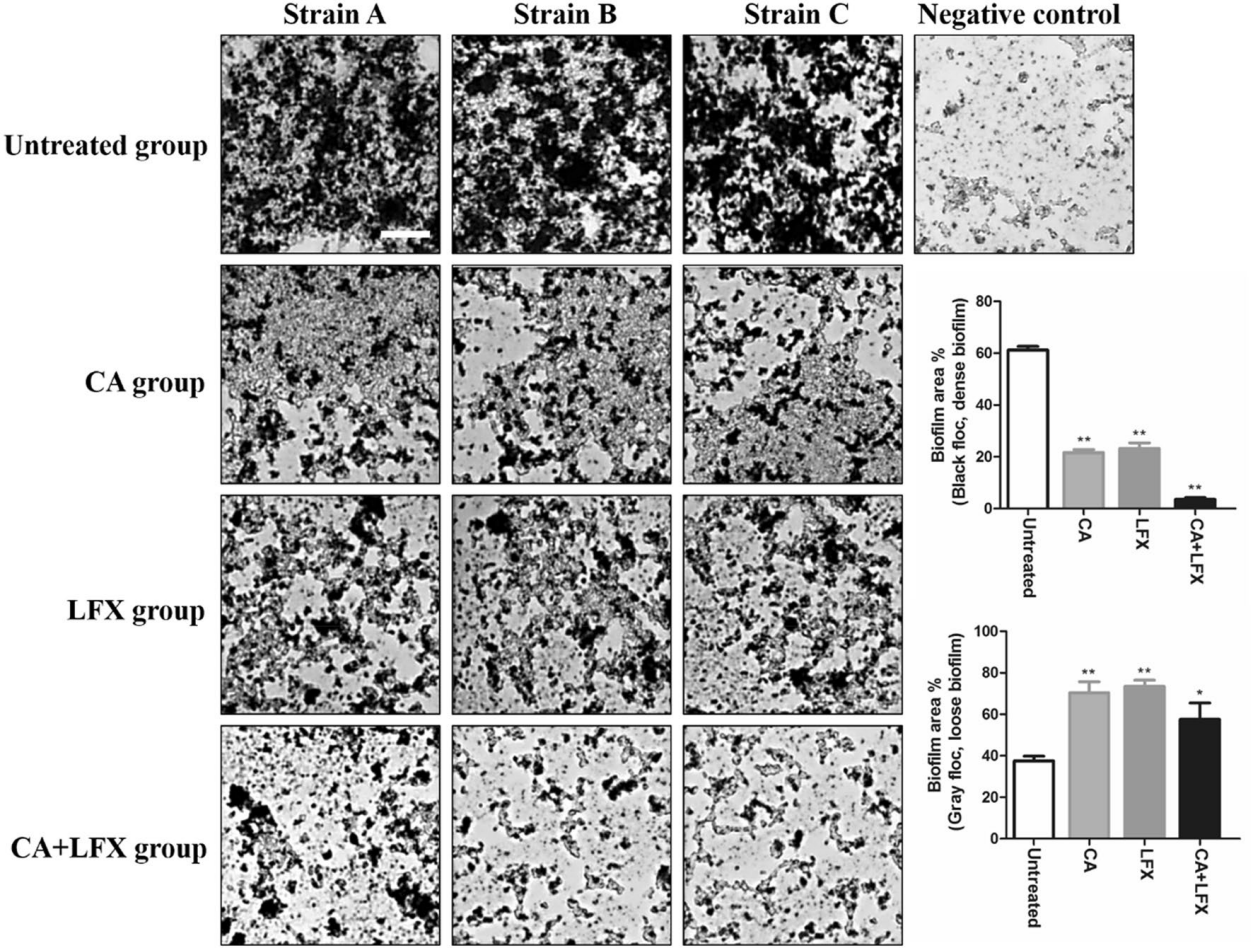
The biofilm formation (floccule) in each group in KPN strain A, strain B and strain C. The image were captured by optical microscope (× 400, scale bar 50 μm). The statistic of the average biofilm area ratio are expressed as mean ± SD for each group, the data was repeated by three independent experiments. *p < 0.05, **p < 0.01 *vs* untreated group. *CA* Chlorogenic acid, *LFX* Levofloxacin.

Under the optical microscope, scattered black spots can be seen in the whole field of vision. If there is no gray black flocculent, it is judged as negative result; if there is thick gray black flocculent covered, with many layers, dense, it is judged as positive result. The gray black flocculent is biofilm, black is dense biofilm, and gray is loose biofilm. In the result of the study, we found that the strains of A, B and C in the untreated group were all observed the thick gray black flocculent; the flocculent was obviously thinned in CA or LFX single administration; While massive black spots were observed in CA and LFX combined administration, and flocculent was rare. In the negative control group, only scattered black spots were seen (Fig. 3).

The statistical data showed that the proportion of black flocs (representing dense biofilm) in the single drug group was less than 25%, that in the combined drug group was less than 5%, and that in the untreated group was about 60% (Fig. 3). Importantly, the area of black floccules (dense biofilm) in CA group and LFX group was significantly smaller than that in untreated group (*p* < 0.01), and the area of gray floccules (loose biofilm) was significantly larger than that in untreated group (*p* < 0.01), which proved that the area of biofilm in CA group and LFX group was thinner than that in untreated group. There was no significant difference in the area of black floccules (dense biofilm) and gray floccules (loose biofilm) between CA group and LFX group. The results showed that the inhibition effect of the two drugs on the formation of biofilm was the same at the concentration of 1/2 MIC. While the area of black floccules (dense biofilm) in CA and LFX combination group was significantly smaller than that in CA group and LFX group (*p* < 0.01), and the area of gray floccules (loose biofilm) was also smaller than that in CA group and LFX group (Fig. 3). It was proved that the combination of CA and LFX had better effect in inhibiting biofilm formation than that in single use.

### The mortality of KPN infected mice

**Figure 4:**
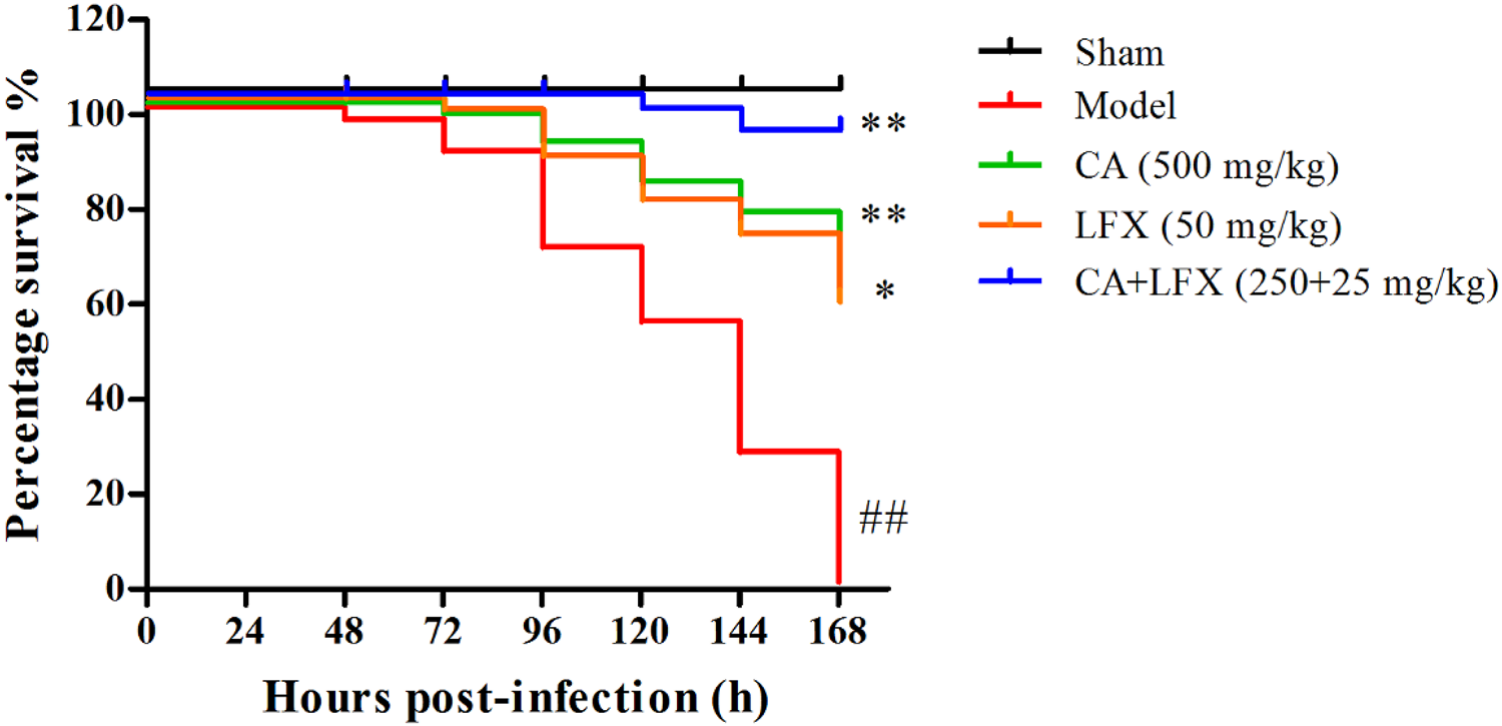
The survival curve of the mice after KPN infection in each group (n = 12). ^##^*P* < 0.01 *vs* sham group, *p < 0.05, **p < 0.01 *vs* model group. *CA* Chlorogenic acid, *LFX* Levofloxacin.

After 24 h of KPN inoculation, the symptoms of the mice showed faster respiration, lower activity, disordered bristle or coat, and increased secretion around the eyes. After 48 h of KPN inoculation, mice began to die, and all mice were died within 168 h (7 days) in the model group, as shown in Fig. 4, indicating that KPN can induce severe pulmonary inflammatory response. CA treatment group, LFX treatment group and the combination of CA and LFX treatment group were obviously reduced the mortality rate of KPN infected mice, compared with the model group. In addition, the combination of CA and LFX treatment group was also significantly increased the survival rate of mice, compared with CA and LFX single treatment group (Fig. 4).

### The effect of CA, LFX and CA + LFX on serum levels of inflammation factors

**Figure 5:**
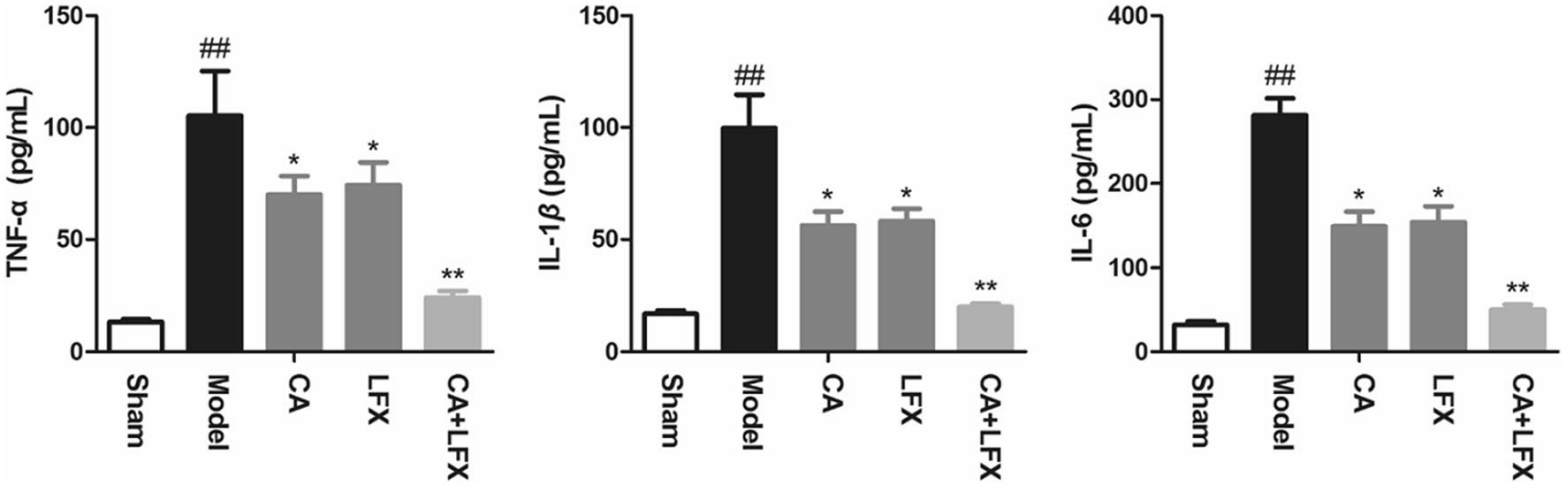
The effect of CA, LFX and CA + LFX on serum levels of TNF-*α*, IL-1*β* and IL-6. Data are expressed as mean ± SD for each group (n = 12). The experiment was repeated by three independent experiments. ^##^*p* < 0.01 *vs* sham group, *p < 0.05, **p < 0.01 *vs* model group. *CA* Chlorogenic acid, *LFX* Levofloxacin.

In Fig. 5, the result showed that the levels of tumor necrosis factor-alpha (TNF-*α*), interleukin-1*β* (IL-1*β*) and interleukin-6 (IL-6) in serum after 48 h of KPN infection in mice. From Fig. 5, we can see that the levels of TNF-*α*, IL-6 and IL-8 significantly increased in model group, compared with the sham group (*p* < 0.01). The result indicated that the mice have been displayed organism inflammation. While after CA, LFX and CA + LFX treated, the levels of the inflammation factors were significantly decreased, compared with the model group, especially in the CA and LFX combination group (*p* < 0.05, *p* < 0.01).

### Histopathological analysis of lung tissue

**Figure 6:**
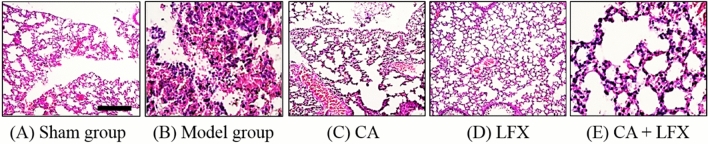
Histopathological changes of mice lung in each group (× 200, scale bar 100 μm). (**A**) Sham group; (**B**) Model group; (**C**) CA (500 mg/kg); (**D**) LFX (50 mg/kg); (**E**) CA + LFX (250 + 25 mg/kg) group. *CA* Chlorogenic acid, *LFX* Levofloxacin.

After 48 h of KPN inoculation, the lung tissue of the dissected mice was congested and edematous, and the millet like abscess or nodule can be observed on the surface of lung tissue. Under the light microscope, there are normal structure of lung tissue in Sham group (Fig. 6A). While there were a lot of neutrophil infiltration, fibrin exudation, capillary congestion, partial alveolar wall rupture, and obvious pathological manifestations of acute inflammation of lung tissue in KPN infected mice (Fig. 6B). The degree of the pathological injury was significantly reduced in CA, LFX, and CA + LFX treatment group, especially in CA and LFX combination group (Fig. 6C–E).

### Effect of CA, LFX and CA + LFX on protein expression of inflammation pathway in lung tissue

**Figure 7:**
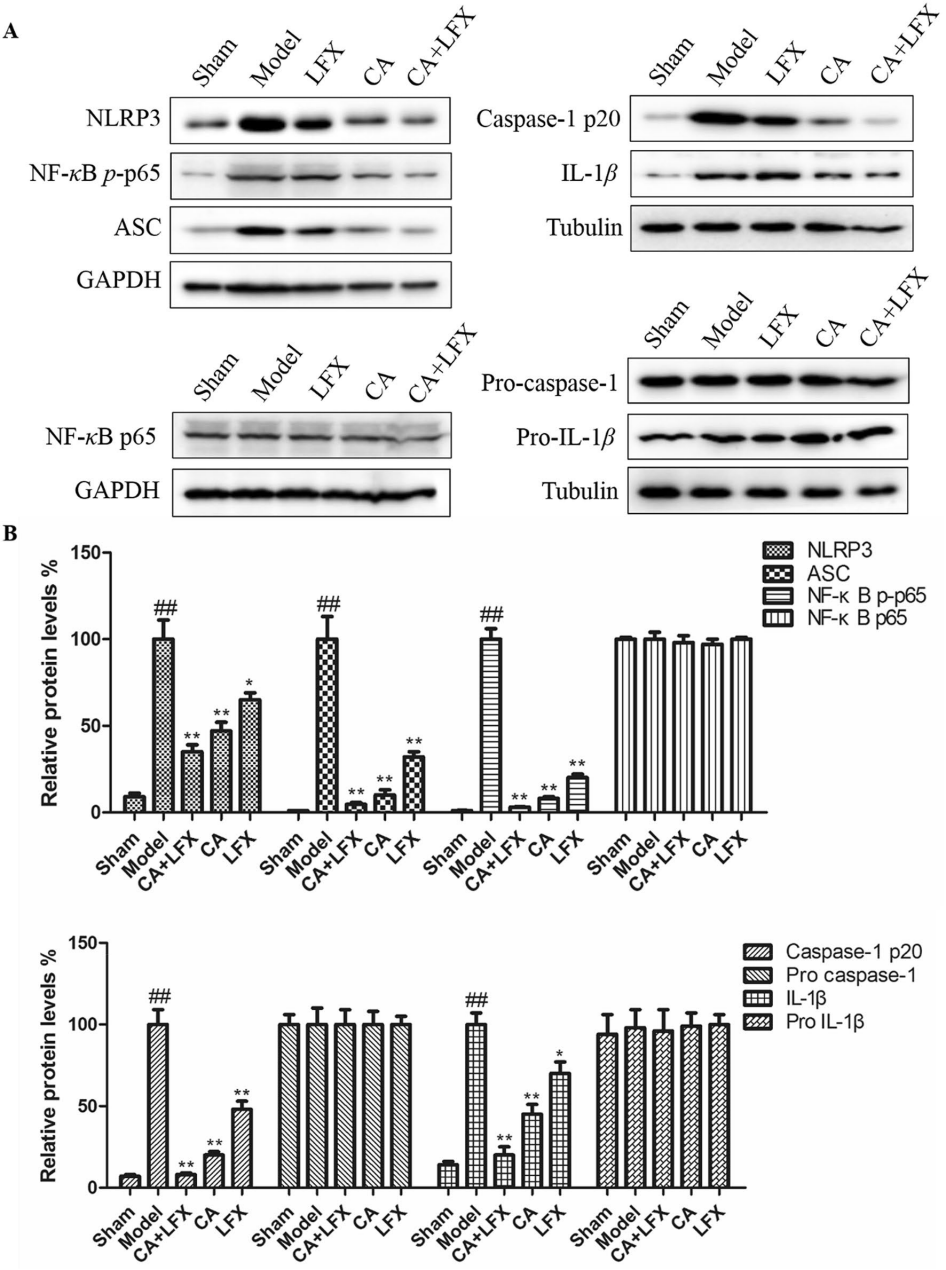
Protein expression of inflammation pathway in lung tissue. (**A**) The protein expression of NLRP3, ASC, NF-*κ*B p-p65, Caspase 1 p20 and IL-1*β* in lung tissue of all group mice. (**B**) The statistical data of each protein expression in each group. The values are given as the mean ± S.D. (n = 12). The experiment was repeated by three independent experiments. ^##^*p* < 0.01 vs Control; *p < 0.05, **p < 0.01 vs Model. *CA* Chlorogenic acid, *LFX* Levofloxacin. Full-length blots are presented in Supplementary Figures.

Many studies have confirmed that KPN infection can trigger a variety of inflammatory pathways. In our study, we found that after KPN infection, the protein expression of NLRP3, ASC, phosphoration NF-*κ*B p65 (NF-*κ*B *p*-p65), IL-1*β* and caspase 1 p20 was significantly increased in lung tissue, compared with the Sham group (Fig. 7). In addition, CA, LFX and CA + LFX treatment group were dramatically decreased the above protein expression, compared with the model group, especially in the group of CA and LFX combination (Fig. 7). The result indicated that CA can decrease the KPN infected lung inflammation, and CA can also increase the effect of LFX, which exhibited a synergic effect.

## Discussion

KPN is the main pathogen of community and hospital infection, which can cause respiratory tract infection, intraperitoneal infection, urinary system infection, blood flow infection and other infectious diseases^36,37^. The emergence of multi drug resistance, broad durability and even pan drug resistance KPN brings great difficulties to clinical treatment, but also increases the length of stay and medical costs of patients^38^. In recent years, it has been found that the formation of biofilm is one of the reasons for the pathogenicity and drug resistance of many bacteria^39^. Compared with planktonic bacteria, it has stronger environmental adaptability, can resist phagocytosis, evade host immunity, and is resistant to a variety of antibiotics, which makes the clinical treatment of biofilm related infection very difficult^40^.

KPN is one of the bacteria easy to form biofilm, which is one of the main reasons for its drug resistance. It has been shown that it can reach 10–1000 times of bacterial resistance, greatly increasing the difficulty of treatment^40^. Biofilm and drug resistance are two important survival abilities of KPN^41^. In our study, there is 13 strains were biofilm positive in 62 KPN strains, accounting for 21% (13/62), slightly higher than the previous report in our hospital. Recently, it has been reported that KPN with biofilm is more likely to produce ESBLs, which is one of the reasons why it is easy to produce drug resistance. In our study, the result showed that among the collected 62 KPN strains, 20 strains were ESBLs biofilm positive, accounting for 32% (20/62) of the total KPN strains.

The traditional Chinese medicine of clearing away heat and detoxifying has low price, low side effects, and is not easy to lead to bacterial drug resistance^42^. It has unique advantages in the prevention and treatment of bacterial infection. From many years of clinical trials, CA has been found to be an effective component of traditional Chinese medicine with a wide antibacterial spectrum^20^. It has been shown that CA can inhibit the biofilm formation of Pseudomonas aeruginosa, but the inhibition effect on KPN is not reported. In this study, we observed the inhibition of CA combined with LFX on the biofilm of KPN in vitro, which laid a foundation for further study in vivo, and provided experimental basis for clinical antibacterial strategy against multi drug resistant KPN. The results showed that CA with a concentration of 512 μg/mL could effectively inhibit the formation of KPN biofilm in vitro. More importantly, we found that the combination of CA and LFX had a good synergistic effect on inhibiting the formation of biofilm, which exhibited that CA can play a certain synergistic effect on quinolones in the process of inhibiting KPN in vitro, which may improve the sensitivity of multidrug-resistant bacteria to quinolones, make them kill bacteria to the maximum extent and reduce the production of drug resistance.

In addition, in our study, we established the KPN infection mice model with the screened ESBLs biofilm positive strain, and investigated the antibacterial ability of CA and CA in combination with LFX on KPN infected mice in vivo. The result showed that CA could significantly reduce the symptoms of KPN infection mice, and CA + LFX significantly reduced the mortality and inflammatory response of mice. After 48 h of KPN infection, the serum levels of TNF-*α*, IL-1*β* and IL-6 increased significantly. While CA treatment group significantly reduced the levels of TNF-*α*, IL-1*β* and IL-6, especially in CA + LFX group, compared with the model group.

Furthermore, many studies have confirmed that KPN infection can trigger a variety of inflammatory pathways. However, there are few studies on the effect of KPN infection on NLRP3. NLR is a pattern recognition receptor family which is widely expressed in the cytoplasm. NLRP3 (NLR pyrin domain-3) is one of the most deeply studied NLR families^43^. The activation of NLRP3 inflammasomes requires two signals participation: the first is that bacteria or viruses bind to toll like receptor (TLR4) on the cell surface, activate NF-*κ*B, and promote the production of pro-IL-1*β* and pro-IL-18 precursor; the second signal is that it can promote NLRP3/ASC (apoptosis associated speck-like protein)/pro-caspase-1 protein complex assembly, when stimulated by PAMP and DAMP. Pro-caspase-1 self-shear to the activation form caspase-1, caspase-1 will cut pro-IL-1*β* and pro-IL-18 into active forms and secrete them to the extracellular, then activate downstream signal pathway, cause inflammatory reaction^44–48^. In our study, we found that after KPN infection, the protein expression of NLRP3, ASC, NF-*κ*B *p*-p65, IL-1*β* and caspase 1 p20 was significantly increased in lung tissue, compared with the Sham group. While CA treatment group can markedly decrease the above protein expression, and CA can also have a synergic effect with LFX in KPN infected mice.

In this study, the single component of traditional Chinese medicine CA was used in combination with quinolones, which provided the experimental basis for the combination of traditional Chinese medicine and Western medicine in the antibacterial strategy of KPN with biofilm. In the future, we will expand the sample numbers, and explore the action mechanism in-depth. At the same time, the accumulated operating experience in our experiment also provide help for the colleagues engaged in the research of biofilm.

In summary, the study found that ESBLs biofilm positive strains plays about 32% (20/62) in the collected 62 KPNs from the First People’s Hospital of Yunnan Province. CA can significantly reduce the formation of biofilm in vitro, and also can alleviate the inflammation reaction in KPN infected mice in vivo. Interestingly, the combination of CA and LFX exhibited a synergic effect in vitro and in vivo.

## Methods

### Reagents

Tumor necrosis factor-alpha (TNF-*α*, Ek-M21159), Interleukin (IL)-6 (Ek-M21235) and IL-1*β* (Ek-M20166) ELISA detection kit were purchased from Biotechnology Co., Ltd. Shanghai enzyme research (China). ASC (sc-271054) was purchased from Santa Cruz Biotechnology (USA). Caspase 1 p20 (22915-1-AP) was purchased from Proteintech (USA). Caspase 1 (2225), NF-*κ*B p65 (8242) and phosphor NF-*κ*B p65 (3033) were purchased from Cell Signaling Technology (USA). Horseradish peroxidase (HRP)-conjugated goat anti-mouse IgG, HRP-conjugated goat anti-rabbit IgG antibodies were provided by Proteintech Group, Inc. (USA).

Chlorogenic acid (110,753–200,413, > 99.5) and Levofloxacin (130,537–200,301, > 98.5%) were provided by China Institute of pharmaceutical and biological products inspection (China). KPN 17489 (biofilm negative strain) and KPN 19936 (biofilm positive strain) were obtained from the First People’s Hospital of Yunnan Province.

### KPN source

From June 2018 to January 2019, 62 strains of *Klebsiella pneumonia* were collected from clinical samples of the First People’s Hospital of Yunnan Province (elimination of repeated strains in the same part of the same patient), the written informed consent was taken from all the participants for clinical samples used in the study. These bacteria were identified by VITEK-2 compact automatic bacterial identification drug sensitivity analyzer of BIOMERIEUX Company (France). The purified strain was inoculated into calf serum and frozen at − 20 °C. All the methods were carried out in accordance with the National Institutes of Health Guidelines of China and related human ethical regulations of Yunnan University. All experiments protocols in the study were reviewed and approved by the Institutional Human Ethics Committee of Yunnan University with the approval number of WHSOPR-2017-12.

### Screening of positive biofilm strains^38^

The positive biofilm strains was evaluated by the semi-quantitative crystal violet staining. The operation process of the study was according to the literature of Candan et al. reported^38^. KPN 17489 as the negative biofilm strain, KPN 19936 as the positive biofilm strain.

### Screening of ESBLs strains^49–51^

62 strains of *Klebsiella pneumonia* were resuscitated and transferred to Colombian blood agar plate and MacConkey plate, then cultured at 37 °C incubator for 24 h. The ESBLs strains were analyzed by the VITEK-2 compact automatic bacterial analyzer (BIOMERIEUX, France), and evaluated according to the standards of clinical and laboratory standards institute (CLSI, 2012 version). The bacteriostatic efficacy was detected by Kirby–Bauer (K–B) drug sensitive paper (MERIEUX Company). Ceftazidime (30 μg/tablet) and ceftazidime/clavulanic acid (30/10 μg/tablet), cefotaxime (30 μg/tablet) and cefotaxime/clavulanic acid (30/10 μg/tablet) were selected for the paper. When the diameter of bacteriostatic circle of ceftazidime is less than or equal to 22 mm, the diameter of bacteriostatic circle of cefotaxime is less than or equal to 27 mm, and the difference between the diameter of bacteriostatic circle of any of the two drugs (ceftazidime and cefotaxime) with and without clavulanic acid is more than or equal to 5 mm, ESBLs can be confirmed.

### Minimal inhibitory concentration (MIC) detection^52^

The MIC was determined by broth microdilution according to CLSI guidelines^51^. The CA and LFX were diluted with Mueller–Hinton (MH) broth to 10 gradient concentrations (4096, 2048, 1024, 512, 256, 128, 64, 32, 16, 8 μg/mL). The final concentration of bacterial suspension was 5 × 10^5^/mL, 100 μL/well for 96 well plate, respectively. ATCC 700,603 as the standard KPN control, the treated drugs were incubated for 24 h at 37 °C. The medium in the hole with clear appearance regard as no bacteria growth, MIC is the minimum bacteria growth inhibitory drug concentration.

The MIC of the combination of CA and LFX was determined by the modified agar dilution checkerboard method of Rand et al.^53^. The agar dilution checkerboard method was modified as follows: CA and LFX were diluted into 4 gradient concentrations (CA: 4096, 2048, 1024, 512 μg/mL; LFX: 128, 64, 32, 16 μg/mL), respectively. Add 25 μL each of CA concentrations and 25 μL each of LFX concentration to the rows and columns of 96 well plates, respectively, to form 4 × 4, totally 16 combined drug concentrations. Then add 50 μL, 5 × 10^5^/mL bacteria into each well, cultured at 37 °C for 24 h. The evaluation and calculation of the fractional inhibitory concentration index (FICI) was followed with the rules for checkerboard assay proposed by Odds^53,54^.

### The effect of CA, LFX and CA + LFX on the formation of biofilm^55–57^

The concentration of CA prepared to 512 μg/mL, LFX prepared to 1/2 MIC, CA + LFX was prepared with 1 mL, 512 μg/mL of CA and 1 mL, 1/2 MIC of LFX for each bacteria strain. KPN17489 (biofilm negative strain) as negative control. Add 2 mL of 5 × 10^5^/mL bacteria into each well (the well has been placed with sterile cover glass), then add drugs respectively in each well. Culture at 37 °C for 5 days, change fresh medium every 24 h with drugs. Then take out the cover glass, silver staining and observed under optical microscope.

### Animals

Ninety male C57BL/6 mice (8 weeks age) with body weight 22 ± 2 g were obtained from experimental animal center of Yunnan University. All the mice were free access to food and water, six mice were kept in one polyacrylic cage, and housed in the standard controlled conditions with the temperature in 24 ± 1 °C, the humidity in 55 ± 5% and 12 h day/night cycle. The human care of the mice received was according to the National Institutes of Health Guidelines of China and related ethical regulations of Yunnan University. All the mice were quarantined for one week before the experiments. At the end of the experiment, the mice were fasted for 12 h before sampling of material. The animal work was carried out at Yunnan University. All experiments related animals in the manuscript were reviewed and approved by the Institutional Animal Care and Use Committee on the Ethics of Animal Experiments of Yunnan University.

### Experimental design

After 1 week quarantined, ninety male mice were randomly divided into five groups (n = 18): Sham group, Model group, CA group (500 mg/kg), LFX group (50 mg/kg), and CA + LFX group (250 + 25 mg/kg). All C57BL/6 mice were anesthetized by inhaling isoflurane. Then the mice were in the position of head up and upright, and KPN suspension was dripped into the nasal cavity for 30 μL (10^8^ CFU/mL). The mice in Sham group were dripped with the same volume of normal saline by the same method. After inoculation, the mice kept their heads upright for 20 s to ensure that KPN suspension or normal saline could enter both lungs evenly due to gravity. When the mice woke up, they were placed in the cage to eat freely. After 30 min of KPN inoculation, CA group (500 mg/kg), LFX group (50 mg/kg), and CA + LFX group (250 + 25 mg/kg) were administrated by oral gavage respectively. The Sham and model group were administrated by oral gavage with the same volume of normal saline. After 48 h of drug administration, collect the blood from mice ophthalmic vein plexus for detection of biological indexes, and randomly select six mice in each group for lung histopathological examination and inflammation related protein expression detection. Observe and record the survival rate of mice within one week. The animals were sacrificed by cervical dislocation.

### Drug combination of CA and LFX on biofilm formation

Take the colonies of the three strong biofilm positive strains (strain A, strain B and strain C), and prepare them into 0.5 Maxwell’s turbidity with normal saline, respectively. LFX was diluted with LB broth to 1/2 MIC of each strain (1/2 MIC of strain A, B and C was 16, 32 and 16 μg/mL respectively), CA was prepared as 512 μg/mL. The biofilm negative strain KPN17489 was taken as negative control. Put 2 mL diluted drugs (CA, LFX and CA + LFX) into 6-well plates (sterile cover glass was placed in each well) respectively, the untreated group was added 2 mL LB broth instead the drugs, the negative control was also added 2 mL LB broth. Added 100 μL bacteria liquid (strain A, strain B and strain C) to each well, the negative control was added 100 μL KPN17489, cultured at 37 °C for 5 days, and change the medium with fresh drugs every 24 h. Then take out the cover glass and carry out silver staining. First, rinse the cover glass with sterile normal saline for 3 times, fix it in 2.5% glutaraldehyde PBS solution for 1 h, and wash with distilled water for 1 min. Then combine with saturated calcium chloride solution for 15 min, wash with distilled water for 1 min, and react with 5% AgNO_3_ solution for 15 min. Next, develop color with 1% hydroquinone solution for 2 min, rinse with distilled water for 1 min, fix with 5% sodium thiosulfate for 2 min, and wash with distilled water for 1 min. After drying, observed and imaged the biofilm formation under optical microscope.

The image of the biofilm formation of each group was analyzed by the color histogram analysis of Photoshop software, the black flocculent represent dense biofilm, the grey flocculent represent loose biofilm.

### The serum levels of TNF-*α*, IL-1*β* and IL-6 assay

The serum levels of TNF-*α*, IL-1*β* and IL-6 were measured according to the protocol provided by commercial ELISA kit from the manufacture of Biotechnology Co., Ltd. Shanghai enzyme research (China). The observation absorbance of TNF-*α*, IL-1*β* and IL-6 was read at 450 nm and the data are expressed as pg/mL.

### Histopathological observation of mice lung tissue

After 48 h of the drug administration, randomly select six mice in each group to collect the lung tissue for lung histopathological examination. Weight and cut about 1 × 1 × 1 cm lung specimen from the largest lung lobes of the right lung. Fixed the lung specimens in 4% neutral formaldehyde buffer for overnight, then embedded in paraffin, cut into 5 µm thickness for hematoxylin and eosin (H&E) staining. Finally, examined and photographed the H&E sections under Olympus BX-50 light microscope at 200 × magnification.

### Protein expression detection of inflammation-related proteins in lung tissue by Western blot

The protein expression of ASC, Caspase 1 p20, Caspase 1, NF-*κ*B p65 and phosphor NF-*κ*B p65 in lung tissues were analyzed by Western blot. First washed the lung tissue with pre-cold phosphate buffer saline (PBS) buffer to remove congestion, then homogenized with the pre-cold tissue homogenizer in homogenate buffer (50 mmol/L, pH 7.5 Tis-HCl, 150 mmol/L NaCl, 1 mmol/L phenyl methyl sulfonyl fluoride, 1 mg/mL aprotinin, 4 mg/mL leupeptin) on ice bath. Centrifuge the homogenate at 4 °C, 10,000 rpm/min for 5 min, and collect the supernatant. Protein concentration was quantified by BCA kit. Load 40 μg proteins to 10% SDS-PAGE electrophoresis gel for running at 125 V for about 2 h, then transferred proteins from gel to PVDF membrane at 4 °C, 100 V for 1.5 h. First antibody dilution ratio is 1:1000, second antibody dilution ratio is 1:5000. Protein membranes were exposured and imaged at Bio-Rad imager (Bio-Rad ChemiDoc MP, USA) with ECL (Enhanced chemiluminescent substrate for horseradish peroxidase (HRP), Thermo Fisher Scientific) reagent for 1 min.

### Statistical analysis

The biological data presented as mean ± SD, the statistical comparisons were followed by One-way ANOVA analysis of Dunett’s t-test with GraphPad Prism 6.0 software (URL: https://www.graphpad.com/). The statistic and curve comparison of the animal survival rate was determined by log-rank test of Kapplan-Meyer analysis with GraphPad Prism 6.0 software (URL: https://www.graphpad.com/). *p* < 0.05 and *p* < 0.01 showed a statistically significant.

## Supplementary information

Supplementary Information.
