# Author Correction: Synergistic effect of chlorogenic acid and levofloxacin against *Klebsiella pneumonia* infection in vitro and in vivo

**DOI:** 10.1038/s41598-025-91502-1

**Published:** 2025-03-06

**Authors:** Shirui Tan, Jing Gao, Qingrong Li, Tieying Guo, Xiangshu Dong, Xuehui Bai, Jinghui Yang, Shumei Hao, Feifei He

**Affiliations:** 1 School of Agriculture, Chenggong Campus, Yunnan University, South Section, East Outer Ring Road, Chenggong District, Kunming, 650500 People’s Republic of China; 2 Center for Life Sciences, School of Life Sciences, Yunnan University, Kunming, 650500 People’s Republic of China; 3 Dehong Tropical Agriculture Research Institute of Yunnan, Ruili, 678600 People’s Republic of China; 4 School of Life Sciences, Yunnan Normal University, No.1, Yuhua Area, Chenggong District, Kunming, 650500 Yunnan People’s Republic of China; 5 The Second Affiliated Hospital of Kunming Medical University, Kunming, 650101 People’s Republic of China; 6 Department of Paediatrics, The First People’s Hospital of Yunnan Province, The Affiliated Hospital of Kunming University of Science and Technology, 157 Jinbi Road, Kunming, 650032 People’s Republic of China; 7 Yunnan Clinical Medical Center for Hematological Diseases, The First People’s Hospital of Yunnan Province, 157 Jinbi Road, Kunming, 650032 People’s Republic of China

Correction to: *Scientific Reports* 10.1038/s41598-020-76895-5, published online 17 November 2020

The original Article contains errors. Due to an error during figure assembly, in Figure 3 the panels of Strain C “LFX group” and “CA + LFX group” show images that are partially overlapping with the panels from the same conditions from Strain B. Both affected panels of Strain C have been replaced. The updated Figure 3 is shown below.

**Figure 3:**
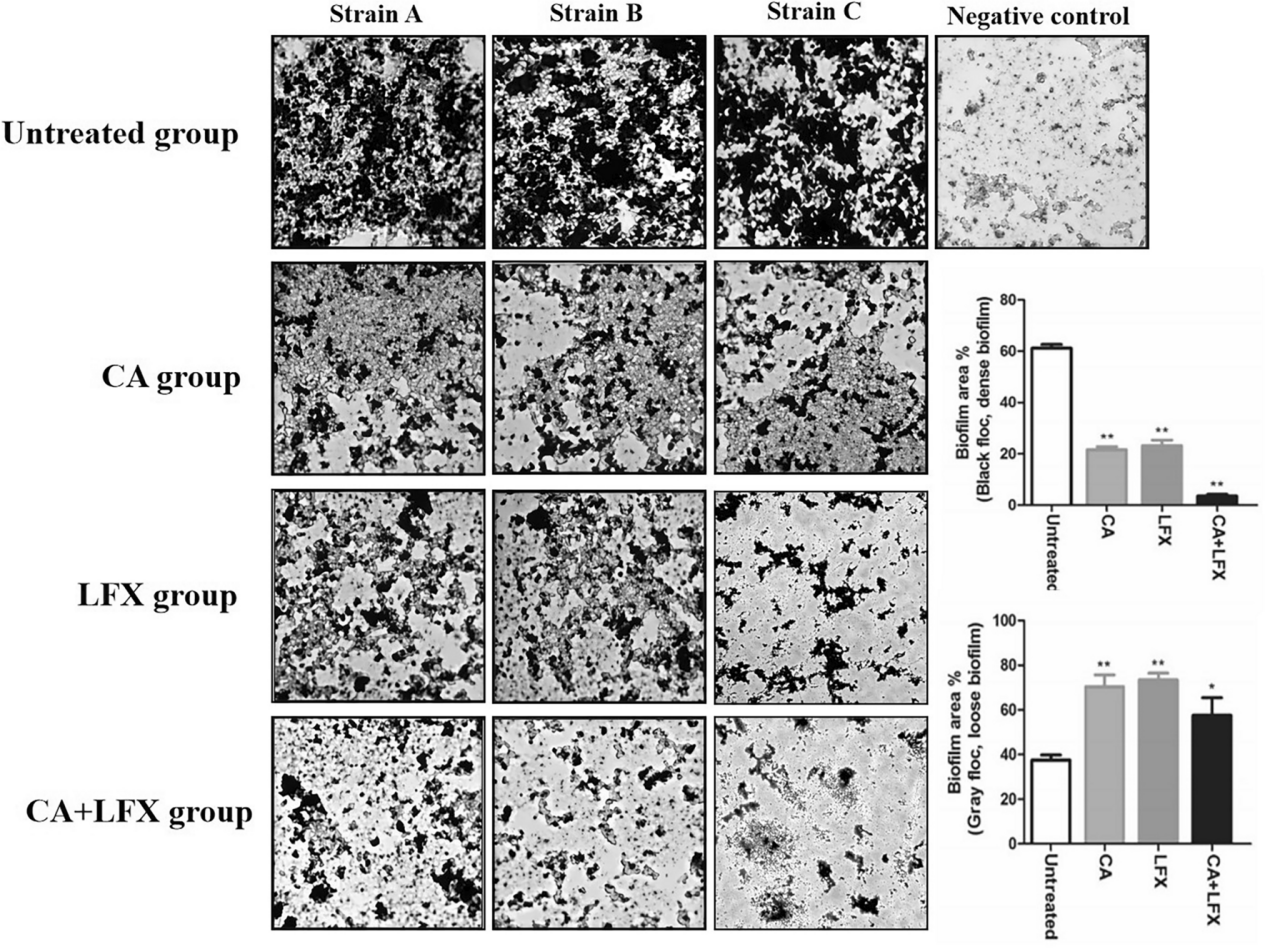
The biofilm formation (floccule) in each group in KPN strain A, strain B and strain C. The image were captured by optical microscope (× 400, scale bar 50 μm). The statistic of the average biofilm area ratio are expressed as mean ± SD for each group, the data was repeated by three independent experiments. *p < 0.05, **p < 0.01 *vs* untreated group. *CA* Chlorogenic acid, *LFX* Levofloxacin.

