# Supplementary material for: Synergistic effect of chlorogenic acid and levofloxacin against Klebsiella pneumonia infection in vitro and in vivo
Source: Sci Rep. 2020 Nov 17;10:20013. doi: 10.1038/s41598-020-76895-5 (PMC7672055; doi:10.1038/s41598-020-76895-5)
Supplement: Supplementary file 1 — Supplementary Information. [file 41598_2020_76895_MOESM1_ESM.docx]

**Supplementary Information**

# Synergistic effect of chlorogenic acid and levofloxacin against *Klebsiella pneumonia* infection in vitro and in vivo

Shirui Tan^1, 2#^, Jing Gao^1#^, Qingrong Li^5#^, Tieying Guo^3^, Xiangshu Dong^1^, Xuehui Bai^3^, Jinghui Yang^6*^, Shumei Hao^4*^, Feifei He^1*^

*^1^ School of Agriculture, Yunnan University, Kunming 650500, P.R. China*

*^2^* *Center for Life Sciences, School of Life Sciences, Yunnan University, Kunming 650500, P.R. China*

*^3^ Dehong Tropical Agriculture Research Institute of Yunnan, Ruili 678600, P.R. China*

*^4^ School of Life Sciences, Yunnan Normal University, Kunming 650500, P.R. China*

*^5^ The Second Affiliated Hospital of Kunming Medical University, Kunming 650101, P.R. China*

*^6^ The First People’s Hospital of Yunnan Province, The Affiliated Hospital of Kunming University of Science and Technology, Kunming 650101, P.R. China*

^#^ The author contributed equally.

^*^ The author are the co-correspondence author.

**Correspondence to:**

**Jinghui Yang**, Department of paediatrics, The First People’s Hospital of Yunnan Province, 157 Jinbi Road, Kunming 650032, P.R. China. Tel: +86871-63638643; E-mail: [yangjh1029@126.com](mailto:yangjh1029@126.com).

**Shumei Hao**, School of Life Sciences, Yunnan Normal University, No.1, Yuhua Area, Chenggong District, Kunming, Yunnan, China, 650500, P.R. China, E-mail: [haosm@sina.com](mailto:haosm@sina.com).

**Feifei He**, School of Agriculture, Chenggong Campus, Yunnan University, South Section, East Outer Ring Road, Chenggong District, Kunming, China Tel: +86-871-65031539; Fax: +86- 871-65031539; E-mail: feifeihe163@163.com


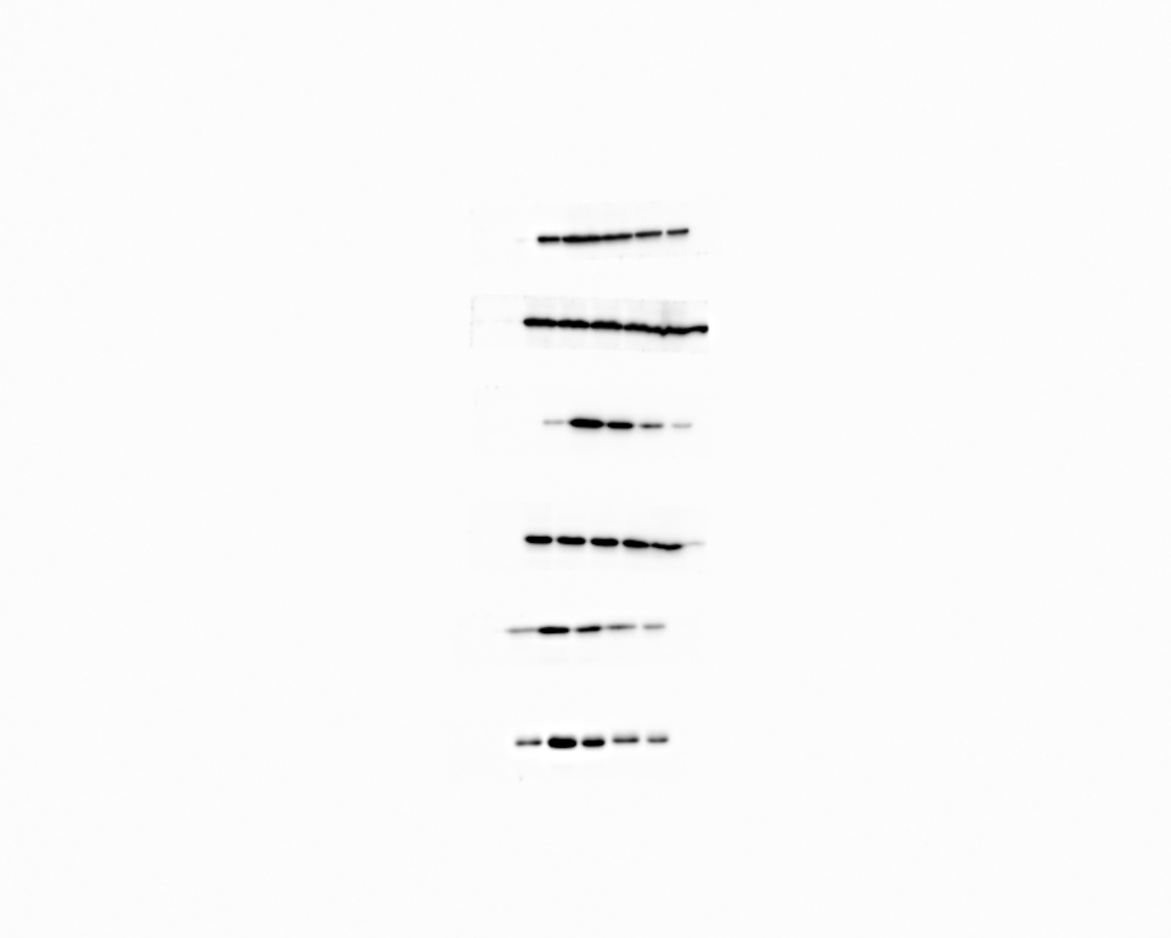


Figure S1. Uncropped western blots of GAPDH, GAPDH, Caspase-1 p20, Pro-caspase-1, ASC and NLRP3 from up to down


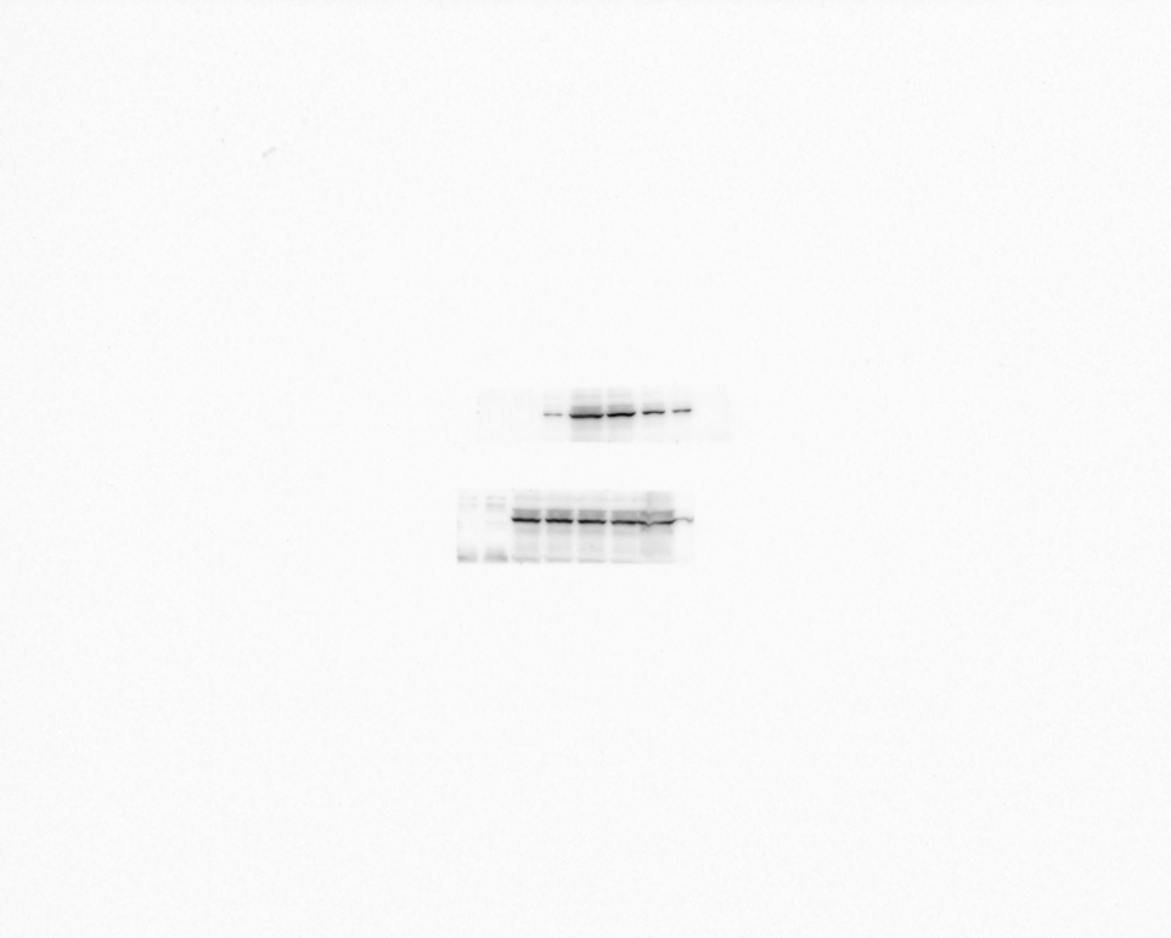


Figure S2. Uncropped western blots of NF-*κ*B *p*-p65 and NF-*κ*B p65 from up to down


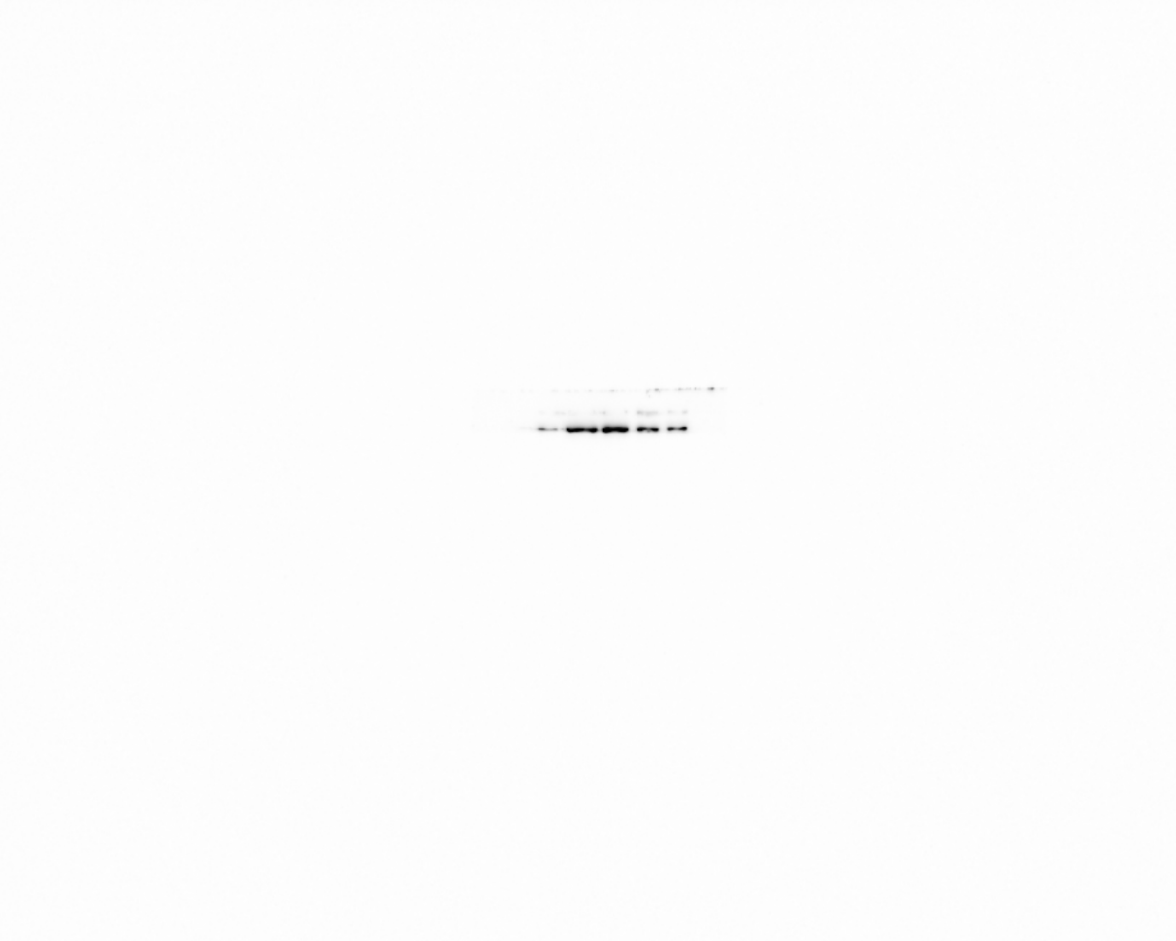


Figure S3. Uncropped western blots of IL-1*β*


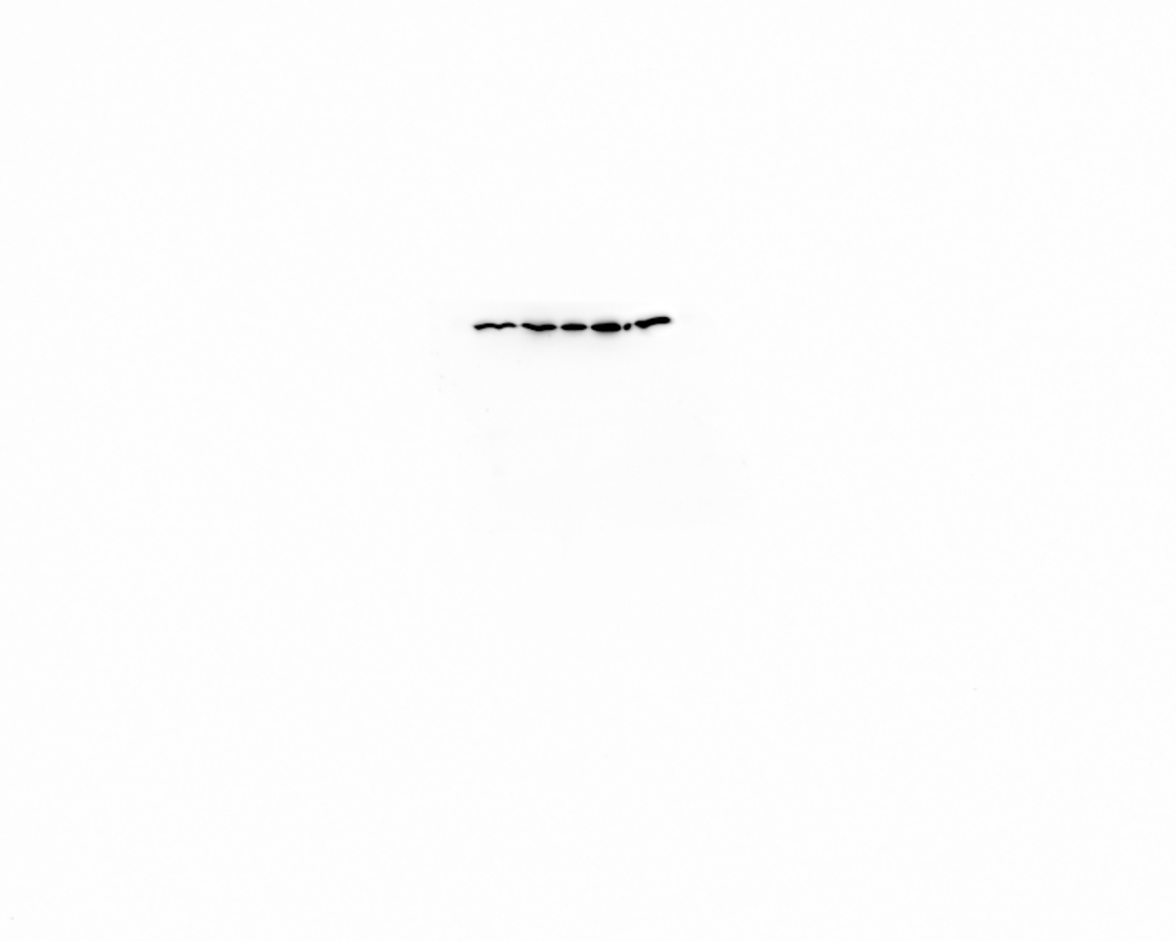


Figure S4. Uncropped western blots of Pro-IL-1*β*


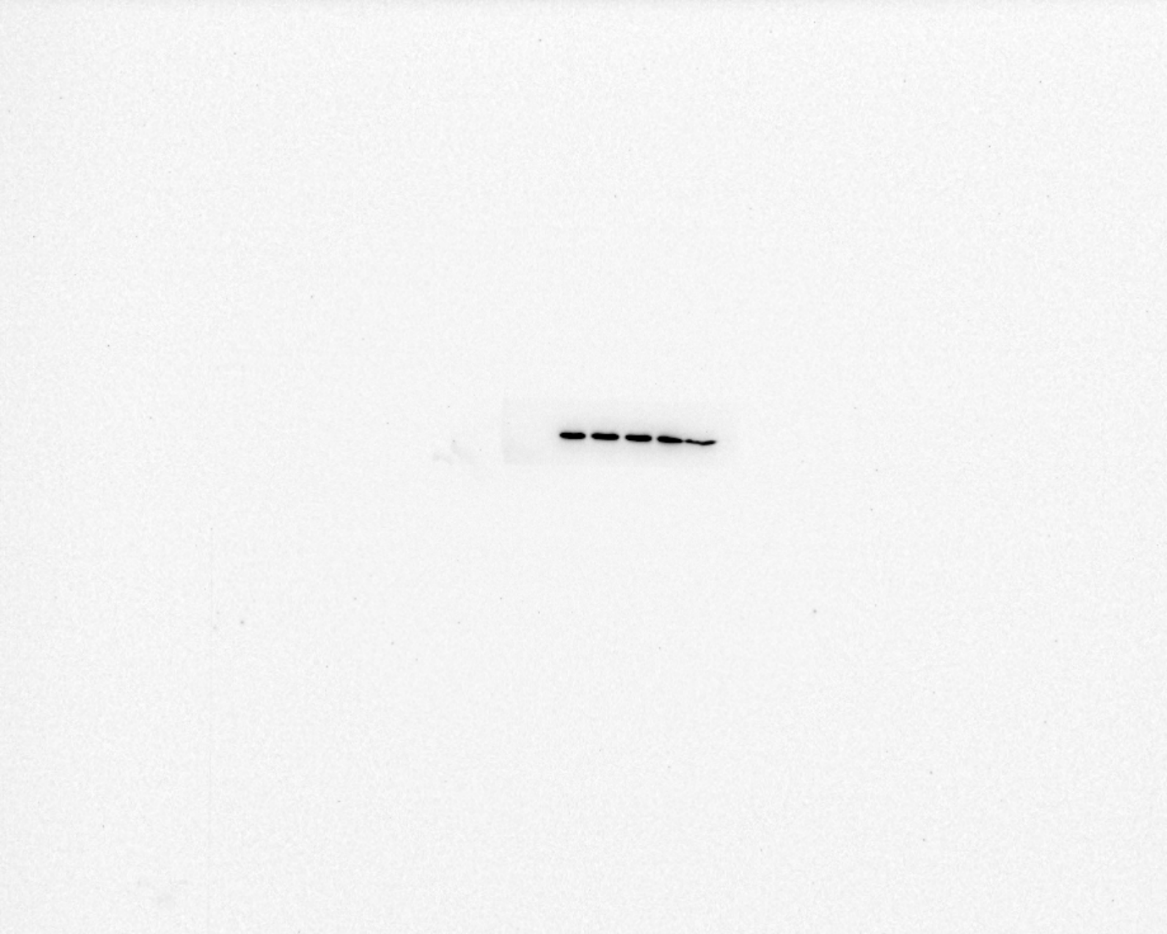


Figure S5. Uncropped western blots of Tubulin


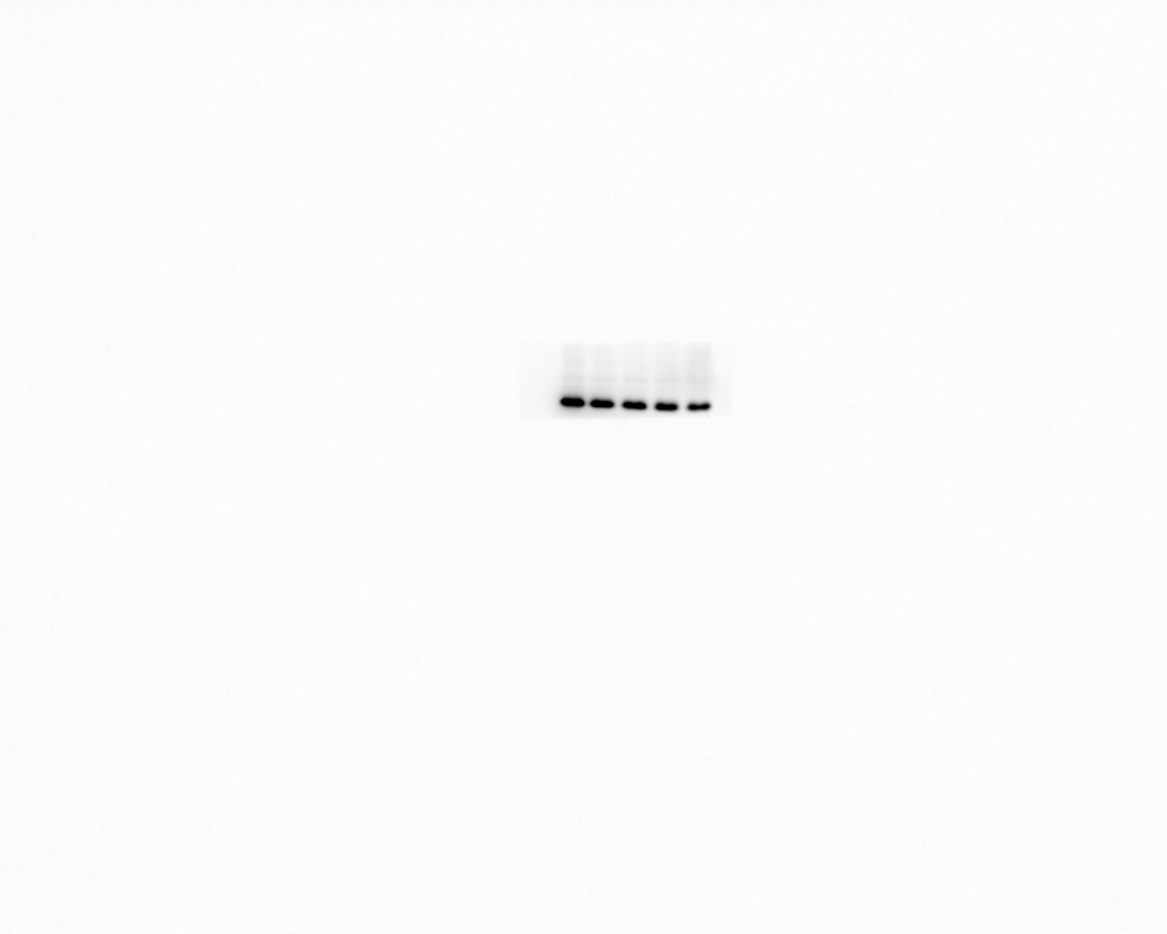


Figure S6. Uncropped western blots of Tubulin
